# Regulatory interplay between soybean root and soybean cyst nematode during a resistant and susceptible reaction

**DOI:** 10.1186/s12870-014-0300-9

**Published:** 2014-11-25

**Authors:** Parsa Hosseini, Benjamin F Matthews

**Affiliations:** School of Systems Biology, George Mason University, Manassas, VA USA; Computational Biology Branch, National Center for Biotechnology Information, National Institutes of Health, Bethesda, MD USA; Soybean Genomics and Improvement Laboratory, United States Department of Agriculture, Beltsville, MD USA

**Keywords:** Soybean, Soybean cyst nematode, SCN, Transcription factor binding site

## Abstract

**Background:**

Plant–parasitic nematodes (PPNs) are obligate parasites that feed on the roots of living host plants. Often, these nematodes can lay hundreds of eggs, each capable of surviving without a host for as long as 12 years. When it comes to wreaking havoc on agricultural yield, few nematodes can compare to the soybean cyst nematode (SCN). Quantifying soybean (*Glycine max*) transcription factor binding sites (TFBSs) during a late–stage SCN resistant and susceptible reaction can shed light onto the systematic interplay between host and pathogen, thereby elucidating underlying *cis–*regulatory mechanisms.

**Results:**

We sequenced the soybean root transcriptome at 6 and 8 days upon independent inoculation with a virulent and avirulent SCN population. Genes such as *β*–1,4 glucanase, chalcone synthase, superoxide dismutase and various heat shock proteins (HSPs) exhibited reaction–specific expression profiles. Several likely defense–response genes candidates were also identified which are believed to confer SCN resistance. To explore magnitude of TFBS representation during SCN pathogenesis, a multivariate statistical software identified 46 over–represented TFBSs which capture soybean regulatory dynamics across both reactions.

**Conclusions:**

Our results reveal a set of soybean TFBSs which are over–represented solely throughout a resistant and susceptible SCN reaction. This set furthers our understanding of soybean *cis*–regulatory dynamics by providing reaction–specific levels of over–representation at 6 and 8 days after inoculation (dai) with SCN.

**Electronic supplementary material:**

The online version of this article (doi:10.1186/s12870-014-0300-9) contains supplementary material, which is available to authorized users.

## Background

Obligate parasites, such as plant–parasitic nematodes (PPNs), are infamously known for their ability to suppress host defense mechanisms and cripple yield of many agricultural crops. Such devastation is tightly orchestrated by nematode effector proteins that commandeer host–plant metabolic machinery. One of the most destructive PPNs to soybean yield is the soybean cyst nematode (SCN; *Heterodera glycines*). Worldwide, approximately 1.5 billion dollars in soybean yield is lost annually due to SCN infestations [1,2]. In SCN susceptible soybeans, this devastation begins when the female juvenile–stage 2 (J2) nematode penetrates the host root. J2 effector proteins are injected into the root, dissolving plant cell walls and driving formation of a metabolically–active, multinucleated feeding site known as a syncytium [3]. Newly–molted J3 males and females feed from this nutrient–rich syncytium, subsequently molt into J4 larvae and copulate [4]. After approximately 30 days post–copulation, a hardened sac of SCN eggs known as a cyst becomes visible to the naked–eye. In the resistant reaction however, cysts are not visible since J2 nematodes can neither form a nutrient–rich syncytium nor copulate. Thus, J2 nematodes starve to death.

With next–generation sequencing (NGS) now becoming a central assay in transcriptomics, entire transcriptomes can now be sequenced at unprecedented resolution. Fueled by the economic impact of SCN infestations, numerous studies have utilized NGS assays to sequence and quantify the soybean transcriptome [5-8].

In this study, we extend such works by conducting transcriptomic and regulatory analyses on soybean roots (Peking cv.) inoculated with SCN. We sequence the soybean root transcriptome and contrast resistant and susceptible SCN reactions at 6 and 8 days after inoculation (dai). Our findings reveal likely defense–response gene candidates and a potential regulatory “signature” that captures TFBS over–representation throughout both resistant and susceptible reactions.

## Results and discussion

### Illumina sequencing and read alignment

cDNA libraries from soybean roots were generated after independently inoculating roots for both 6 and 8 dai in two SCN populations, NH1-RHg (confers resistant reaction in Peking; Race 3) and TN8 (confers susceptible reaction in Peking; Race 14). A baseline control cDNA library was also created from roots uninoculated with SCN. RNA was prepared using the Illumina TruSeq sample preparation kit. Single–end RNA–sequencing (RNA–Seq) was performed on the Illumina GAIIx, producing a total of 30 million reads 80 bp in length. Across all sequenced libraries, quality assessment subtracted between 10%—19% of reads for being either a contaminent sequence or of low quality (Table 1). Using the BWA aligner [9], quality reads were mapped against the soybean transcriptome build version 1.1 [10]. Reads aligning to multiple transcripts were identified and assigned to the transcript with the highest quality score. In total, 59% to 69% of quality–assessed reads mapped to the soybean transcriptome.

**Table 1:** **Soybean–SCN pathogenesis RNA–Seq summary**

**SCN population**	**Time point**	**SRA**	**Reads**	**Filtered**	**Aligns to soybean**
Uninoculated	Control	SRR849499	2,141,303	401,913 (19%)	1,201,664 (69%)
Race3	6 dai	SRR847313	8,069,844	1,130,372 (14%)	4,640,251 (67%)
	8 dai	SRR848922	7,319,342	745,019 (10%)	4,135,793 (63%)
Race14	6 dai	SRR848921	9,160,690	1,624,774 (18%)	4,486,182 (59%)
	8 dai	SRR849498	4,078,344	637,475 (15%)	2,193,208 (63%)
Total	–	–	30,769,523	4,539,553 (14%)	16,657,098 (63%)

Summary of reads generated throughout a Race 3 and Race 14 SCN inoculation. Low quality reads were subtracted from the total read–set. Remaining reads were then mapped to the soybean transcriptome.

### Soybean transcript abundance and profiling during SCN pathogenesis

Differential expression tests were performed using the R package DESeq [11]. Soybean transcripts were functionally annotated using both Gene Ontology (GO) [12] and PFAM [13]. Both fold change and *log*_2_ fold change of expression profiles (as RPKM) were computed between experimental and uninoculated samples. To render a soybean transcript differentially expressed (DE), the transcript had to have a log_2_ fold change greater than or equal to ±1.0 and have atleast 5 mapped reads across all replicates. A total of 12,377 soybean transcripts were identified to be DE in at least one of the samples (Additional file 1). To disseminate the plant–pathogen defense–response landscape, a subset of 181 DE transcripts were mined and classified given their GO and PFAM functional annotations (Table 2, Additional file 2). Interestingly, virtually all of these annotation classifications exhibited induced expression profiles exclusive to the resistant reaction. For instance, all 12 transcripts of *β*–1,4–glucanase (*β*–1,4–G) were generally induced throughout the resistant but suppressed in the susceptible reaction. Numerous studies reveal how a pathogenic nematode can commandeer not only *β*–1,4–glucanase but other cellulases to drive formation of a nematode feeding site [14-16]. Both Tucker et al. [16] and Ibrahim et al. [14] quantified this destructive commandeering capability by quantifying the soybean transcriptome using high–throughout microarrays. This latter study, though examining soybean–root knot nematode interplay, reveals cell–wall modeling, defense response, and metabolism, to be the most impacted host pathways following pathogenic nematode infection. Critical genes encoding isoflavonoid and flavonoid biosynthesis such as chalcone synthase (ChS), chalcone reductase (ChR), and chalcone isomerase (ChI) also exhibited similar induced expression profiles. Glutathione S-transferase (GST) genes were also induced in the resistant reaction. GST is a class of enzymes involved in reactions leading to xenobiotic degradation [17], and has been shown to be induced during an SCN resistant reaction [18-20].

**Table 2:** **Various genes perceived during defense response are expressed during SCN inoculation**

		**Median** ***l*** ***o*** ***g***_**2**_ ** RPKM**			
		**Race 3**		**Race 14**	
**Function**	***n***	**6 dai**	**8 dai**	**6 dai**	**8 dai**
*β*–1,4–G	12	1.14	0.85	0.27	0
4CL	26	0.46	0.69	-1.03	-0.27
A–8 LOX	18	0	1.03	-1.77	-0.59
ChR	5	1.07	1.01	0	-0.53
ChI	6	1.33	0.63	-0.37	-0.62
ChS	15	1.18	1.39	-0.76	-0.73
GST	21	1.15	1.14	-1.02	0
GLY I	5	1.41	1.11	-1.49	-1.39
L–13S LOX	17	0.91	1.40	-1.76	-0.81
PCS	4	0.74	1.49	-1.29	-0.41
PR5	15	1.66	0.54	-1.38	-0.53
PR10	15	1.31	1.12	-1.16	-1.23
PDI	9	1.08	1.60	-0.89	-1.08
RnDR	5	1.39	0	0	0
SOD	8	1.12	0.60	-0.58	0

Numerous genes are involved in defense–response. DE transcripts were binned based on GO or PFAM annotated function, yielding bins of differing size, *n*.

Transcripts of genes encoding two lipoxygenase (LOX) gene family members, arachidonate 8-lipoxygenase (A–8 LOX; EC: 1.13.11.40) and linoleate 13S-lipoxygenase (L–13S LOX (LOX2); EC: 1.13.11.12) were also induced throughout both 6 dai and 8 dai resistant reactions. The role A–8 LOX plays during a nematode reaction has yet to be elucidated, however lipoxygenases in–general are consistently induced throughout a resistant SCN reaction [21-24]. This raises speculation that A–8 LOX may be perceived during SCN pathogenesis.

Ribonucleoside-diphosphate reductase (RnDR; EC: 1.17.4.1) and protein disulfide-isomerase (PDI; EC: 5.3.4.1) were induced in the resistant reaction. Both RnDR and PDI are thioredoxins, a family of reductases known to play defense–response roles upon perception of a pathogen [25-27]. Little is known about the role RnDR plays in SCN pathogenesis, however an earlier microarray study examined abaxial and adaxial soybean embryo expression profiles upon exposure to auxin 2,4-dichlorophenoxyacetic acid (2,4–D). Microarray results revealed differentially expressed levels of RnDR 21 days after auxin inoculation [28]. PDI on the other hand, is a well–studied thioreductase expressed during plant defense [29,30], especially in soybean roots undergoing a resistant SCN reaction [31].

Pathogenesis–Related (PR) transcripts, namely PR5 and PR10, were induced in the resistant reaction. PR genes were expressed not just during SCN nematode pathogenesis [32-38] but also throughout abiotic stress [39], phytohormone signaling [40] and drought [41].

Glyoxalase I (GLY I; lactoylglutathione lyase, EC: 4.4.1.5) was also induced throughout the resistant reaction. GLY I has been shown to exhibit an induced expression profile in pumpkin seeds exposed to numerous abiotic stresses [42]. Lastly, little is known about the role phytochelatin synthetase (PCS) plays throughout SCN pathogenesis, however PCS has been shown in a prior study to be induced during aphid herbivory [43].

Following quantification of the SCN–inoculated soybean root transcriptome, our analyses support earlier works by Klink et al. ([44,45]), Kandoth et al. ([20]), and Li et al. ([33]). We build–on such studies by identifying a small subset of potentially novel defense–response candidate genes as well as a biologically–sound proximal regulatory landscape that captures host–SCN pathogenesis interplay.

### Gene Ontology enrichment in resistant and susceptible reactions

To identify statistically significant Gene Ontology (GO) annotations, the top 750 induced and 750 suppressed genes across for all SCN samples each independently underwent GO Process enrichment using the AgriGO server [46]. Numerous GO Processes were statistically significant across resistant and susceptible reactions (Table 3). GO Process *p*–values were adjusted using Bonferroni False Discovery Rate (FDR) and all GO Processes with adjusted *p*–values less than 0.05 were selected.

**Table 3:** **Abundance of enriched Gene Ontology annotations**

		**Race 3**		**Race 14**	
		**6 dai**	**8 dai**	**6 dai**	**8 dai**
Count	Induced	53	48	25	19
	Suppressed	73	104	113	86

Enriched GO annotations throughout each inoculation. Per inoculation, the top–750 induced and top–750 suppressed DE transcripts were identified and enriched GO annotations were identified. Only enrichments with a Bonferroni–corrected *p*–value less than 0.05 were selected. Counts represent both GO Process and GO Function.

The top 30 most statistically significant GO Processes within induced genes were identified (Table 4). Processes such as “defense response”, “syncytium formation”, “response to other organism”, “response to oxidative stress”, and “response to stress”, were revealed to be statistically significant mainly in the resistant reaction when compared to the susceptible. Processes associated with organelle modification and intracellular organization also exhibited similar reaction–specific significance. This race–exclusivity exposes the crucial role basal operations play during pathogen perception.

**Table 4:** **GO Process enrichment of induced soybean genes**

		**−** ***l*** ***o*** ***g***_**10**_ ***F*** ***D*** ***R***	
**Term**	**Description**	**Race 3**	**Race 14**
GO:0042545	Cell wall modification	10.49	0
GO:0042547	Cell wall modification during multidimensional cell growth	10.52	4.25
GO:0044085	Cellular component biogenesis	3.20	0
GO:0034622	Cellular macromolecular complex assembly	4.25	0
GO:0046916	Cellular transition metal ion homeostasis	0	4.52
GO:0031497	Chromatin assembly	11.74	0
GO:0006333	Chromatin assembly or disassembly	10.18	0
GO:0006325	Chromatin organization	7.18	0
GO:0051276	Chromosome organization	6.11	0
GO:0006952	Defense response	6.69	1.45
GO:0006323	DNA packaging	11.55	0
GO:0065003	Macromolecular complex assembly	3.92	0
GO:0051704	Multi-organism process	3.69	0
GO:0009825	Multidimensional cell growth	6.08	1.79
GO:0006334	Nucleosome assembly	12.92	0
GO:0034728	Nucleosome organization	11.85	0
GO:0006996	Organelle organization	3.48	0
GO:0010117	Photoprotection	4.56	0
GO:0009828	Plant-type cell wall loosening	8.40	2.56
GO:0009827	Plant-type cell wall modification	8.56	0
GO:0009831	Plant-type cell wall modification during multidimensional cell growth	6.38	2.02
GO:0009664	Plant-type cell wall organization	6.25	0
GO:0065004	Protein-DNA complex assembly	12.40	0
GO:0009725	Response to hormone stimulus	2.50	5.95
GO:0051707	Response to other organism	5.21	1.88
GO:0006979	Response to oxidative stress	10.66	0
GO:0006950	Response to stress	5.35	0
GO:0006949	Syncytium formation	7.45	2.43
GO:0055076	Transition metal ion homeostasis	0	4.52
GO:0006414	Translational elongation	5.16	0

GO Process enrichment from the top 750 induced transcripts. Numerous GO Processes associated with cell–wall modification, intracellular organization and defense response exhibit increased enrichment during the resistant reaction.

Similarly, the top 30 most statistically significant GO Processes within suppressed genes were also identified (Table 5). Contrasting GO Processes in suppressed genes to that of induced genes reveals an entirely different catalog of annotations. For instance, 20 of the 30 GO Processes in suppressed genes are statistically significant across both resistant and susceptible reactions. This indicates that nematode effectors are generally operable in a race–independent manner and capable of effortlessly suppressing a majority of crucial basal processes.

**Table 5:** **GO Process enrichment of suppressed soybean genes**

		**−** ***l*** ***o*** ***g***_**10**_ ***F*** ***D*** ***R***	
**Term**	**Description**	**Race 3**	**Race 14**
GO:0006066	Alcohol metabolic process	0	2.52
GO:0016051	Carbohydrate biosynthetic process	4.56	7.92
GO:0044262	Cellular carbohydrate metabolic process	0	2.17
GO:0043094	Cellular metabolic compound salvage	2.88	5.53
GO:0006091	Generation of precursor metabolites and energy	83.18	87.31
GO:0006544	Glycine metabolic process	2.20	0
GO:0006096	Glycolysis	1.48	3.95
GO:0018130	Heterocycle biosynthetic process	6.42	4.20
GO:0019318	Hexose metabolic process	1.79	5.33
GO:0042743	Hydrogen peroxide metabolic process	2.66	0
GO:0006555	Methionine metabolic process	2.12	0
GO:0006740	NADPH regeneration	0	2.37
GO:0006733	Oxidoreduction coenzyme metabolic process	0	2.92
GO:0009853	Photorespiration	6.48	9.04
GO:0015979	Photosynthesis	215.70	211.61
GO:0009765	Photosynthesis, light harvesting	81.37	68.25
GO:0009768	Photosynthesis, light harvesting in photosystem I	52.95	39.57
GO:0019684	Photosynthesis, light reaction	132.78	130.48
GO:0009767	Photosynthetic electron transport chain	43.33	47.11
GO:0009773	Photosynthetic electron transport in photosystem I	23.73	28.55
GO:0042549	Photosystem II stabilization	4.76	9.29
GO:0046148	Pigment biosynthetic process	8.81	11.14
GO:0042440	Pigment metabolic process	14.26	17.96
GO:0018298	Protein-chromophore linkage	51.69	42.96
GO:0043467	Regulation of generation of precursor metabolites and energy	1.88	4.38
GO:0042542	Response to hydrogen peroxide	0	5.20
GO:0010035	Response to inorganic substance	0	6.25
GO:0009416	Response to light stimulus	11.30	13.85
GO:0009314	Response to radiation	10.71	13.19
GO:0000302	Response to reactive oxygen species	0	3.73

GO Process enrichment from the top 750 suppressed transcripts. Almost all GO Processes were suppressed in a race–independent manner. The suppressive cocktail of SCN effectors are revealed in the down–regulation of processes associated with photosynthesis, metabolism and biosynthesis.

The most suppressed GO Processes were “photosynthesis”, “photosynthesis, light harvesting”, “photosynthesis, light reaction”, and “generation of precursor metabolites and energy”. Interestingly, it has been shown in prior studies that PPNs can suppress photosynthesis in tomato plants by disrupting cytokinin and gibberellin signaling [47,48]. Aside from photosynthetic processes, those associated with metabolism and biosynthesis were highly suppressed across both reactions. This suggests that both resistant and susceptible SCN populations share a common goal of crippling basal metabolic machinery and suppressing the host machinery responsible for photosynthesis.

### Derivation of over–represented TFBSs

The 1,000 most induced and 1,000 most suppressed genes were identified for each sample and the promoter sequence 2 kb upstream from each genes transcription start site was retrieved and appended to a FASTA file (Additional file 3). To quantify abundance of *cis*–regulatory TFBSs within promoter sequences, we used a collection of 68 plant Position Weight Matrices (PWMs) from AthaMap [49] and JASPAR [50]. PWMs are multi–dimensional matrices frequently used to model regulatory elements, namely TFBSs. Each cell in a PWM represents a weight as to the likelihood a particular base at a specific index is a regulatory element. Thus, mapping PWMs onto promoter sequences and statistically quantifying its abundance reveals insight into the magnitude of TFBS over–representation. To efficiently execute such mapping, we had developed a multivariate statistical software named Marina [51]. Marina maps TFBS models such as PWMs onto promoter sequences and infers magnitude of TFBS over–representation using 7 knowledge–discovery metrics. The Iterative Proportional Fitting (IPF) algorithm [52] normalizes output produced from each of the 7 metrics, enabling unanimous agreement across the metrics as to the magnitude of TFBS over–representation. IPF scores range from 1 to *N* whereby *N* is the total number of over–represented TFBSs. Scores in the range of 1 represent over–represented TFBSs while scores in the range of *N* represent highly under–represented TFBSs.

**Figure 1:**
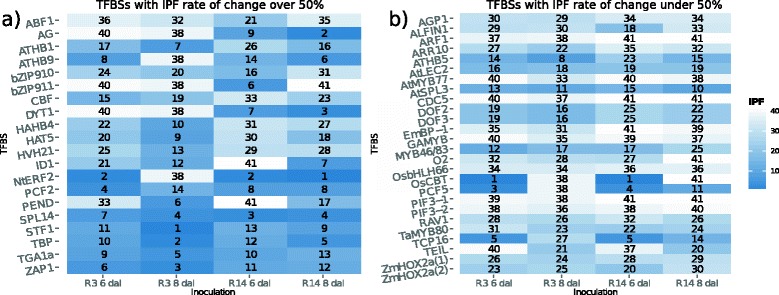
**A heatmap of Marina IPF scores.** Across the four SCN samples, over–represented TFBSs were identified given promoter sequences from the 1,000 most induced and 1,000 most suppressed genes. In total, 46 TFBSs were over–represented in one of the inoculations and 29 TFBSs were over–represented across all inoculations. IPF scores range from 1 to *N* whereby 1 represents over–represented TFBSs and *N* represents under–represented TFBSs. **(a)** Enriched TFBSs within Race 3 and Race 14 reactions with IPF scores having percent difference of at least 50%. **(b)** Enriched TFBSs within Race 3 and Race 14 reactions with IPF scores having percent difference less than 50%.

For all SCN samples, Marina mapped all 68 plant PWMs onto promoter sequences of both induced and suppressed genes. In total, 46 TFBSs were over–represented in atleast one of the four samples (Figure 1). To reveal which TFBSs exhibited variations in their IPF scores, we computed the percent change of IPF scores across both Race 3 and Race 14 timepoints. The difference in Race 3 and Race 14 percent change was derived and partitioned into 2 bins: TFBSs with a Race 3 and Race 14 IPF score percent difference of at least 50% (Figure 1a), and TFBSs with a Race 3 and Race 14 IPF score percent difference under 50% (Figure 1b). Thus, such computation allows for identification of which TFBSs vary greatly not with respect to 6 dai or 8 dai, but with respect to Race 3 and Race 14 inoculations.

There were 29 TFBSs over–represented across all four samples (Additional file 4). If a TFBS was not over–represented in a specific sample, that TFBS was assigned an score of *N*+1 so as to serve as a proxy for being highly under–represented.

### Many TFBSs are over/under–represented in both resistant and susceptible reactions

Contrasting TFBS IPF scores across samples reveals that 30 of the 46 TFBSs either increase or decrease in IPF score regardless of the reaction (Figure 1). For instance, the TFBS for STF1 exhibits a relatively modest increase in its IPF score across both reactions. Interestingly, STF1 IPF score increases from 11th to 1st from 6 dai to 8 dai respectively in the resistant reaction. Besides the role STF1 plays in plant development [53], little is known of the role this transcription factor plays in plant defense.

IPF score for the HAHB4 TFBS greatly increased in the resistant reaction and susceptible reaction. A prior study found HAHB4 to contribute to jasmonic acid and ethylene signaling crosstalk [54]. Similarly, TFBSs for DOF2 and DOF3 exhibited relatively weak increases in IPF scores across resistant and susceptible samples. DOF transcripts have not been explicitly quantified as–far as their gene expression during SCN pathogenesis, however such proteins have been detected during auxin signaling [55]. In contrast to DOF2 and DOF3, the TFBS for TEIL had a near–50% jump in IPF scores across both reactions. Being the tobacco homolog of ethylene insensitive (EIN3), TEIL gene products have been shown to bind directly to the promoter sequence of PR1a, a central contributor in plant defense dynamics [56]. Interestingly, across both resistant and susceptible reactions, TEIL scores appear to be relatively equal to one another.

The *A. thaliana* MYB77 homolog, AtMYB77, exhibits a mild change in IPF score across both resistant and susceptible reactions. Across both reactions, AtMYB77 IPF scores were generally under–represented at 6 dai but become slightly over–represented at 8 dai. An earlier study revealed interaction between MYB77 and auxin response factor 7 (ARF7) [57], further accentuating the role AtMYB77 could play in host–pathogen interplay [58]. The OsCBT TFBS exhibited pronounced IPF scores across all four treatments. In both the resistant and susceptible reaction, OsCBT was highly over–represented only at 6 dai. It was shown that OsCBT mutants conferred increased pathogen resistance upon inoculation with *Magnaporthe grisea*, revealing that OsCBT suppresses defense response [59].

### Several TFBSs are over–represented in a race–dependent manner

The remaining 16 TFBSs were over–represented in one reaction compared to the other. Such TFBSs can expose novel insight into TFBSs over–representation patterns respective to a specific reaction.

ZAP1, a WRKY1 TFBS [60], appears to be highly over–represented during the resistant reaction but slightly under–represented in the susceptible reaction. Being a WRKY TFBS, it comes as no surprise that enrichment of this TFBS in the resistant reaction captures the need to host a significant, systematic plant defense response. Similarly, PIF3–1 and PIF3–2 were both under–represented during the susceptible reaction however slightly over–represented in the resistant reaction. It has been shown that PIF plays roles in phytochrome signaling [61]. Due to its photomorphogenic regulatory capabilities, Since photosynthetic processes are heavily suppressed within resistant and susceptible reactions (Table 5), such suppression explains why PIF3–1 and PIF3–2 have such severely under–represented IPF scores. Indeed SCN pathogenesis does not only disrupt the photosynthetic machinery but also the plants ability to execute sound phytochrome signaling.

## Conclusions

We used RNA–Seq to sequence soybean whole–root (Peking cv.) at both 6 and 8 dai upon inoculation with a resistant (NH1–RHg; Race 3) and susceptible (TN8; Race 14) population. Contrasting TFBSs over–represented in promoter sequences of DE soybean genes across 6 and 8 dai time points exposed underlying transcriptomic and *cis*–regulatory dynamics within the soybean root during pathogenesis. In–total, over 30 million reads from soybean whole–root was sequenced and differential expression analysis revealed 181 transcripts to be statistically and biologically significant during defense–response. Several viable defense–response gene candidates joined these ranks, including glyoxalase I, arachidonate–8 lipoxygenase, phytochelatin synthetase, and ribonucleoside-diphosphate reductase.

46 TFBSs were rendered over/under–represented across all resistant and susceptible samples. Interestingly, 30 of these TFBSs were either over or under–represented across both reactions. Thus, our results reveal presence of a biologically–sound regulatory “signature” that identifies reaction–specific soybean regulatory patterns during both resistant and susceptible SCN reactions.

## Methods

### Plant procurement and SCN inoculation

Glycine max cv. Peking seeds were surface–sterilized by treating the seeds with 10% bleach (0.6% sodium hypochlorite) for ten minutes, followed by several washes with distilled water. Seeds were planted in sterile sand in 20×20 cm flats. Eight days later, seedlings were gently lifted out of the sand and rinsed clean. Five seedlings for each time point were placed on moistened germination paper in 8×12×3.5 cm plastic trays. The SCN populations NH1–RHg and TN8, were independently harvested from stock plants [62]. Females were crushed with a rubber stopper and eggs were washed through a 250 micron screen and collected on a 25 micron screen. Eggs were rinsed into a small covered tray and left to hatch for three days. J2 stage nematodes were further purified by passing them through a 30 micron cloth into deionized, distilled water and gently centrifuged at 250 relative centrifugal force (RCF) for one minute to concentrate to 2,000 J2/ml. Roots from four plants were inoculated with one ml of inoculum. Roots were covered with a second piece of moistened germination paper and the trays were placed in a larger tray with 0.5 cm water below to add humidity and wrapped in a semi-clear plastic bag for the duration of the time points. Three uninoculated control plants were also placed trays and collected separately. Per plant, four plant roots, following 6 and 8 days after inoculation (dai), were harvested and immediately frozen in liquid nitrogen and ground to a fine powder in a mortar and pestle and stored in microfuge tubes at –80°C until RNA extraction. The fifth root was stained for visualization of nematode infection with acid fuchsin [63]. RNA was extracted at 6 dai and 8 dai by phenol/chloroform and lithium chloride precipitation [64]. RNA was treated with DNase to remove any genomic DNA remaining in the samples. RNA integrity was checked by visualizing the intact 18S and 28S ribosomal bands on an agarose gel and concentrations were measured on a Nanodrop spectrophotometer (Thermo Scientific; Waltham, MA).

### RNA extraction and cDNA isolation

cDNA libraries were prepared using the TruSeq RNA Prep Kit according to the manufacturer instruction (Illumina). Briefly, mRNA was purified from four micrograms of total RNA diluted in fifty microliters of nuclease–free ultra pure water using magnetic beads. Resulting mRNA was fragmented at 94°C for eight minutes. Seventeen microliters of fragmented mRNA was used as template for cDNA synthesis performed by a Superscript II Reverse Transcriptase. Second–strand synthesis was immediately performed and fifty microliters of double stranded DNA was transferred to a new tube and submitted to end repair followed by adenylation of 3’ ends. Once adenylation of 3’ reached completion, adapters containing different indexes were ligated to each library. DNA fragments having adapter molecules on both ends were amplified and enriched. Quantification and quality control were performed by loading one microliter of cDNA libraries on an Agilent DNA–1000 chip and running it on an Agilent Technologies 2100 Bioanalyzer.

### Deep–sequencing and transcriptome quantification

For both NH1–RHg (Race 3) and TN8 (Race 14) reactions, cDNA libraries were sequenced from 8 day old soybean whole–root independently inoculated with SCN at 6 dai and 8 dai. Two biological replicates were sequenced for each inoculation and timepoint. Single–end RNA–sequencing was performed on the Illumina GAIIx at the United States Department of Agriculture (USDA), Beltsville, MD. An uninoculated whole–root single–replicate control was also sequenced using the same sequencing protocol. To remove low quality reads across all sequencing runs, custom bash scripts filtered all reads should its 3’ tail have a quality score of less than 22. To remove contaminent reads, sequences were subtracted if they mapped atleast once to both the Ensembl human genome (Hg19) or the JCVI Microbial Resource [65]. Remaining sequences were mapped to the soybean transcriptome (build 1.1) using BWA [9]. Across all SCN inoculated samples, transcript counts underwent normalization and variance estimation using the DESeq R package. To infer magnitude of differential expression, RPKM was computed for all inoculated and uninoculated samples and $log_{2}\left (\frac {RPKM_{\textit {inoculated}}}{RPKM_{\textit {uninoculated}}}\right)$ was subsequently derived. All transcripts with a *log*_2_ RPKM less than 1 and fewer than 5 mapped reads were rendered not differentially expressed.

### Functional annotation & Gene Ontology (GO) enrichment

Functional annotation comprised of homology–based analysis of all sequences in the Phytozome soybean transcriptome. Of these 73,320 soybean transcriptome sequences, 7,810 sequences were subtracted for being either a scaffold or duplicate sequence. BLASTX [66] aligned the remaining 65,510 query sequences onto all UniProt plant proteins [67]. The top–scoring UniProt function annotation was assigned to the query if it did not contain ambiguous keywords, namely “Hypothetical”, “Uncharacterized” or “Unknown”.

For all samples, soybean Phytozome accessions for the top 750 induced and top 750 suppressed transcripts were identified. Gene Ontology (GO) enrichment on each accession–set was performed using the AgriGO web–server [46]. AgriGO settings were modified to quantify GO annotations using the hypergeometric distribution and Bonferroni *p*–value false–discovery rate (FDR) correction. To measure GO Process statistical significance in both resistant and susceptible reactions, the −*log*_10_*FDR* per GO Process was summed across both 6 and 8 dai time points. Subsequently, the top 30 most statistically significant GO Processes from the top 750 induced and suppressed transcript sets were identified.

## Availability of supporting data

All RNA–Seq FASTQ raw data is available from NCBI SRA. Please refer to Table 1 for such accessions.

## Additional files

Additional file 1
**Differentially expressed transcripts across all inoculations.** A table of 12,377 transcripts that are DE across all four SCN inoculations.

Additional file 2
**Differentially expressed transcripts annotated to be involved in plant defense.** A set of 181 transcripts collectively annotated by GO and PFAM annotations to contribute to plant defense.

Additional file 3
**TFBSs over–represented across all inoculations.** A collection of 46 TFBSs over–represented in atleast one inoculation.

Additional file 4
**Promoter sequences of induced and suppressed transcripts.** FASTA sequences representing promoter sequences of induced and suppressed transcripts following 6 dai and 8 dai with SCN virulent and avirulent populations.

## Abbreviations

4CL: 4–
Coumarate–CoA ligase; A–8 LOX: Arachidonate 8-lipoxygenase
L–13S LOX: Linoleate 13S-lipoxygenase
ChI: Chalcone isomerase
ChR: Chalcone reductase
GST: Glutathione S–
transferase; GLY I: Glyoxalase I
GO: Gene Ontology
PCS: Phytochelatin synthetase
PDI: Protein disulfide–
isomerase; PPN: Plant Parasitic Nematode
PR: Pathogenesis–
related; PWM: Position weight matrix
RnDR: Ribonucleoside-diphosphate reductase
SOD: Super–
oxide dismutase; SCN: Soybean cyst nematode
STF1: Starch–
Free 1; TF: Transcription factor
TFBS: Transaction factor binding site
